# From outrage to action: Efficacy moderates the relationship between anger and prosocial political outcomes

**DOI:** 10.1371/journal.pone.0358939

**Published:** 2026-10-14

**Authors:** Monique Mitchell Turner, Ellen C. Reinhart, Dustin Carnahan, David M. Markowitz

**Affiliations:** Department of Communication, Michigan State University, East Lansing, Michigan United States of America; Yeditepe University, TÜRKIYE

## Abstract

Politically motivated anger has surged among American adults in recent years with few signs of decline. Anger has often been associated with aggression and with violent outcomes. Here, we examined nonviolent mobilizing outcomes of anger; specifically the conditions under which anger is associated with political action and voting intentions. Informed by the Anger Activism Model (AAM; [1]), we analyzed aggregated data from a national rolling cross-sectional survey collected in the five weeks before the November 2024 U.S. presidential election (*N* = 1,049). We measured anger intensity, multiple forms of political efficacy (response, internal, external), commitment to social activism, and voting intention. Analyses revealed that the relationship between anger and prosocial political behavior was contingent on efficacy type and level. Among respondents with low response efficacy, very high anger was associated with greater commitment to activism but lower intention to vote. Among respondents high in these forms of efficacy, anger showed little association with either outcome. External political efficacy did not meaningfully moderate these associations. These results provide partial support for the AAM and indicate that anger’s associations with political outcomes depend not only on its intensity but also on which form of efficacy is salient.

## Introduction

Politically motivated anger has surged among American adults [1,2] and it does not appear to be decreasing. For nearly half a century, American adults report through national surveys feeling mild to extreme anger several times per day [3,4]. Since the 2016 US presidential election, surveys show that anger is rising dramatically with 55% of citizens indicating they *always or often* feel angry when thinking about politics [2]. This is vital information because anger has a powerful influence on judgments, decisions, and behavior [5,6].

Yet, the nature and direction of the relationship between anger and judgments and behaviors is complex [7]. Anger has been associated with counter-productive or antisocial outcomes such as superficial reasoning (i.e., low elaboration, [8,9]), closed mindedness [10], biased evaluation of political arguments [10], a reliance on heuristics [11,12], and tolerance for political violence and anti-democratic behaviors [13]. Averill emphasized that all anger does not lead to aggression [3]; anger has also been associated with prosocial behaviors like nonviolent social activism, deep cognitive processing, and a desire to examine arguments for accuracy [14]; nonetheless the negative stereotype persists.

The Anger Activism Model (AAM) predicts that angering stimuli triggers anger, and that the intensity of angry feelings interacts with individuals’ efficacy beliefs in relation to the direction of judgments and decision making. Specifically, under conditions of high efficacy beliefs, a linear relationship between anger and prosocial outcomes is predicted while under conditions of weak efficacy, this relationship is predicted to be nonmonotonic. The AAM has primarily been tested in highly controlled experimental studies with high internal validity [11,15] but has rarely been tested in organic environments where anger is endemic—as it may be during a controversial presidential election cycle.

The objective of this study was to provide a robust test of the AAM in a field setting. We analyzed aggregate data from a national rolling cross-sectional survey with a national sample of American adults. We collected data weekly during the five weeks prior to the November 2024 Presidential election, concluding on November 5, 2024. We measured primary emotions experienced during the period, political efficacy, intentions to engage in social activism and voting intentions.

## Background and literature review

### Anger

The appraisal paradigm [16] and the cognitive-functional paradigm [17] posit that anger stems from a unique pattern of cognitions after exposure to a goal incongruent event/stimulus, whereas the dimensional model of emotion conceives anger to be a strong, negative and high activation emotion. Adhering to an appraisal paradigm, we believe that anger involves perceiving an event or stimulus as a personal slight—whether through offense, harm, or injury to oneself or someone of personal significance. More broadly, anger emerges when individuals interpret an external event as unjust, harmful, or obstructive to their goals and well-being [17]. Uniquely, anger is sparked when individuals feel confident in identifying both the third-party cause and responsible party, and believe the act was intentional [17].

This appraisal pattern not only generates anger but also reinforces subsequent assessments of certainty and perceived control over the situation [18,19]. Similarly, Affective Intelligence Theory [20] posits that anger activates the disposition system, favoring intuitive responses over deliberate cognitive effort. The extent to which individuals experience anger varies based on factors such as perceived relevance of the outcome, personal significance of the issue, and individual differences in trait anger.

Anger has been associated with a wide range of behaviors [21]. It has been linked to aggression and violence [22], and also outcomes such as heightened attention to important issues [15,23] and social activism intentions. We use prosocial descriptively to denote legal, nonviolent, institutionally sanctioned forms of political participation that can benefit others [24]. This usage refers to the class of behaviors we measured and makes no claim about the moral content of participants’ motives or values, which we did not assess. Antisocial outcomes, such as support for political violence or anti-democratic practices, were not measured here and fall outside the scope of the present analyses. We will also use the term political mobilization to refer to the outcomes predicted here: prosocial activism and voting intentions.

### Anger activism model

Turner’s Anger Activism Model (AAM) provides a framework for understanding when anger is associated with prosocial judgment and decision-making versus disengagement from the angering issue [7]. Drawing from communication and psychological research, the model posits that anger, in conjunction with efficacy beliefs, is associated with motivated actions aimed at addressing social injustices (i.e., political mobilization). Rather than characterizing anger solely as an antisocial force, the AAM highlights its association with individual empowerment, including willingness to challenge injustice, advocate for policy change, and mobilize collective efforts.

The model predicts that anger can serve as a catalyst for political mobilization (i.e., peaceful protest, signing petitions) under specific conditions, particularly when the intensity of anger and perceptions of efficacy are high. When efficacy beliefs are weak, a non-linear relationship between anger and outcomes is predicated such that moderate anger is more likely to result in political mobilization, whereas extreme anger may overwhelm individuals, and is associated with disengagement or aggressive reactions. Conversely, when individuals possess high efficacy beliefs, anger is associated with political mobilization, whether in the form of policy influence, structural change, or cultural transformation, as in Fig 1.

**Fig 1:**
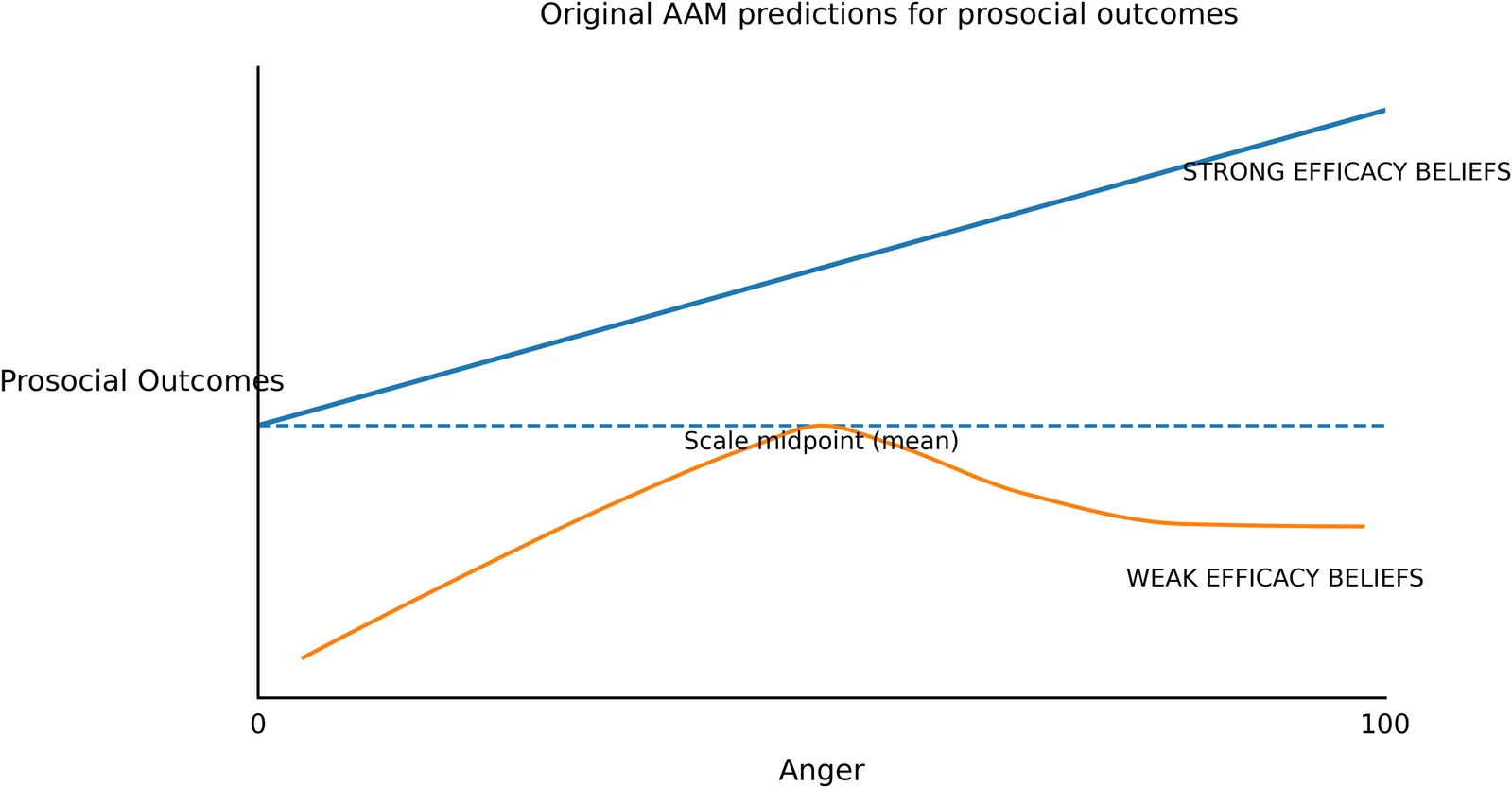
The Anger Activism Model.

A key component of the AAM is perceived efficacy, which consists of two critical dimensions: external or response efficacy, the belief that effective solutions exist for addressing an issue, and internal or self-efficacy, the confidence in one’s ability to contribute to change. Turner emphasized that individuals who perceive themselves as capable of making a difference are more likely to channel their anger in prosocial ways, using it as a source of motivation rather than an impediment to action [7].

Tests of the AAM have shown that under conditions of high efficacy, anger has a positive and linear association with outcomes such as systematic processing (accuracy motivated processing, counterarguing, relevant thoughts, etc.) and activism behaviors (commitment to social activism scale, boycotting, etc.; [11,14,15]). But, under conditions of low efficacy, results are less consistent. Jang et al.’s study showed an inverse-U pattern (as predicted) for accuracy motivations [14]. Turner et al. showed the predicted curvilinear pattern for behavioral intentions [13]. Skurka’s experimental tests of the AAM were not consistent with any two-way interactions between anger and efficacy on attitude outcomes [14,25,26]. That said, Walter et al.’s meta-analysis was mainly consistent with the AAM and they noted the “significant findings concerning anger intensity are particularly intriguing, as they illuminate the importance of further investigation of AAM” [27] (p. 87). One problem that has been noted in this previous experimental research [28] was an inability to manipulate very low efficacy or very high anger. Thus, to some extent, the predictions of the AAM have not been fully tested.

The current study, however, examines anger organically arising throughout a presidential election campaign, which may increase the likelihood of ascertaining data from high (and low) anger participants as well as high and low efficacy. In this context, anger may not stem from a single, isolated incident; rather, it is likely provoked by a broader array of events, communications, and political figures encountered throughout the campaign period. Given this diffuse nature of anger, it is important to consider political efficacy as an additional potential moderating factor. Specifically, this work examines political efficacy, defined as an individual’s belief in their capacity to influence political processes and outcomes, with a focus on external political efficacy, or the perception that political institutions and leaders are receptive to citizen input [29,30]. Higher levels of political efficacy have consistently been linked to stronger political participation, including voting and activism, whereas lower levels are commonly associated with political alienation, mistrust, and disengagement. When people perceive themselves as politically efficacious, they are more inclined to view civic engagement as a meaningful avenue for addressing their concerns, making efficacy a pivotal factor in theories of political behavior and mobilization.

### Anger and political behaviors

Informed by their Affective Intelligence Theory (AIT), Marcus, Neuman, and MacKuen argued that anger activates the “disposition system,” a neural and motivational circuit prompting defense of group norms and engagement with the political environment [31]. Unlike anxiety (which triggers the surveillance system and withdrawal), anger signals that goals are blocked by an identifiable actor, and that action is possible. On this account, anger is associated with greater attention to politics and with mobilization to defend valued positions, both of which are forms of engagement rather than withdrawal. Even so, most political psychology research has emphasized anger’s costs, for example, its association with biased information processing and affective polarization [10,32,33]. Anger has been associated with greater partisan animus, lower openness, and more punitive judgments [34,35]. This large body of work has created a widespread presumption that anger is primarily antisocial or destructive.

Webster’s work epitomizes the dominant focus on anger’s costs [36]. In *American Rage*, he shows that anger is associated with stronger partisan loyalty and identity but also with deeper polarization and eroding trust in outgroups. Similarly, other work by Webster reports that anger is associated with higher political interest and turnout—but typically by amplifying animus, not deliberation or consensus-building [37,38]. Yet, as AIT and other work shows, anger’s approach orientation can also be prosocial, particularly when legitimate channels for redress are visible [39]. The crucial question is not whether anger energizes behavior—it does—but what kinds of behavior it energizes, and under what conditions. This tension is visible in contemporary research on political participation. Van Zomeren, Postmes, and Spears proposed the Social Identity Model of Collective Action (SIMCA), showing that group-based anger about injustice consistently predicts protest, petitioning, and other collective behaviors [40]. Similarly, Iyer, Leach, and Crosby reported that anger—distinct from guilt or shame—was associated with opposition to illegitimate state actions and with mobilization to change unfair policies [41].

This literature positions anger as a response to perceived injustice: when people perceive a wrong, sometimes a wrong experienced by a collective, anger is associated with action on behalf of that collective. This directly challenges the “anger as destructive” paradigm prevalent in political psychology research. While many studies tie anger to bias and aggression, these collective-action models show that anger is associated nonviolent mobilization when efficacy beliefs are present. Without efficacy, anger may be associated with disengagement—outcomes well documented in the political psychology literature [10,35].

Across these traditions, three consistent moderators distinguish when anger is associated with participation versus disengagement. First, anger is associated with participation when people believe that effective solutions exist and that they (or their group) can enact them. Without efficacy, anger is more often associated with cynicism, or with aggression and violence—outcomes heavily documented in political psychology [10,34,35]. Second, integral anger (about the issue), relative to incidental anger (spillover from unrelated provocations), fosters systematic processing and engagement, whereas incidental anger more often produces biased judgments, stereotyping, and hostility [42,43]. This study regards only integral anger. Third, moderate-to-high but manageable anger is associated with prosocial outcomes, while extreme anger has been linked to heuristic reasoning or reactance [44,45], but only when efficacy is very low.

## Study objectives

Several studies have foregrounded anger’s antisocial costs, polarization, bias, aggression, but literature shows that anger can also be a civic resource. Marcus et al. provide the affective architecture: anger as an approach-motivated signal to defend norms [20]. Webster shows that anger is reliably associated with political participation, even as it is also associated with greater animus [37,38]. Van Zomeren et al. and Iyer et al. show anger as a core driver of collective action for justice [40,41]. Together, this work reframes anger from a “destructive force” to a conditional correlate of civic participation: when efficacy is strong, anger is associated with social activism and voting. When efficacy is low or absent, anger is more often associated with disengagement from political behavior. This motivates the present study’s focus on political efficacy as the key moderator of the association between anger and participation.

The present study advances the AAM in four ways. First, it is a field test of a model developed and tested almost exclusively in controlled experiments. Prior AAM research induced anger through a single message about a single issue within a bounded time frame [11,14,15,25,26]; here, anger arose organically over a five-week national election campaign and was diffuse rather than issue-bounded. Whether a model built on induced anger extends to ambient political anger is not a foregone conclusion. Second, this study addresses the type of efficacy and not only its level. The AAM as originally specified treated efficacy as a single construct composed of response and self-efficacy [7]. We measure response efficacy, internal political efficacy, and external political efficacy in the same sample and test each in parallel, which permits a head-to-head comparison that the literature has rarely conducted. Third, we treat activism and voting as distinct outcomes rather than as interchangeable indicators of participation, which allows us to detect divergence between them. Fourth, because the AAM predicts nonmonotonic relationships, we estimate and test nonlinear interactions using average marginal effects and second differences [46] rather than interpreting product-term coefficients, which are not interpretable as interaction effects when the underlying relationships are nonlinear. We therefore predict:

H_1_: Efficacy beliefs moderate the relationship between anger and prosocial outcomes such that when efficacy is high, anger and (a) social activism and (b) probability of voting will be positively and linearly related.
H_2_: When efficacy is weak, there will be a nonmonotonic relationship between anger and (a) social activism and (b) probability of voting.

## Method

### Participants

As part of the Emotional Election Dataset [47], an online survey was administered to U.S. adults from September 30 through November 25, 2024. We partnered with a survey panel company to recruit a sample with demographic characteristics approximating national representativeness across race and ethnicity, gender, and political affiliation. Data collection occurred at a rate of approximately 200 respondents weekly. Given our research focus on the period before the 2024 election, we excluded participants who completed the survey on Election Day (November 5, 2024) or later. The resulting sample (N = 1,049) maintained near-equal representation of the two dominant U.S. political parties (48.4% Democrat, 46.8% Republican, 3.2% Independent, < 1% Other or Prefer not to say). Data collection procedures received approval from the Michigan State University Institutional Review Board (protocol: 11252) before the data collection began. At the start of the survey, participants read about the study and voluntarily agreed to participate by selecting “agree” as their informed consent in place of verbal or written documentation. This study was approved by the Michigan State University Institutional Review Board (STUDY11252). Informed consent was received by all participants. Participants read about the study and agreed to voluntarily participate by selecting “agree,” which served as the informed consent in lieu of written or typed documentation per the ethics committee standards. Informed consent was received by all participants. Study data, materials, and analysis code are available on the Open Science Framework: https://osf.io/n4537.

### Measures

#### Anger intensity.

Participants indicated how much they experienced each of several emotions (i.e., angry, frustrated, anxious, hopeful) by responding to the following question: “You might be feeling more than one emotion. When it comes to the 2024 election season, how much of each of the following emotions are you experiencing?” from 0 *none of this emotion* to 100 *overwhelmingly this emotion*. Anger intensity represents the average of respondents’ response to feeling angry and mad (*M* = 46.99, *SD* = 28.53, *r* = .80***).

#### Efficacy.

Response efficacy. Participants indicated the primary target of their anger by responding to the following question “It is common for people to experience some anger during election season. If you experience anger, what is the main cause of your anger?” and selecting one of the following responses: 1) Donald Trump, 2) Kamala Harris, 3) elected officials in the opposing political party, 4) ordinary people in the opposing political party, 5) mainstream media, 6) social media, 7) my friends and family, 8) a current event (please describe), 9) other (please describe), 10) not applicable. Then, participants responded to the open-ended prompt asking them “Is there anything specific that can be done to address this problem [*primary target of their anger piped in*]?” After, participants then answered the following question: “How confident are you that you can do something to help [*open-ended response about what can be done piped in*]” from 0 *not at all confident* to 100 *overwhelmingly confident.* However, after data collection, we realized that if participants indicated that nothing can be done to address the target of their anger, piping in that text makes rating their confidence in that action unclear (e.g., if someone is certain that nothing can be done, should they rate 0% that nothing can be done or 100% confident that nothing can be done?). To address this, two independent coders rated each open-ended response to identify which stated that “nothing can be done.” For those responses, response efficacy was coded as 0% given that no action could help address the problem. Other open-ended responses that indicated the prompt was not applicable to the participant (e.g., “not applicable I was not angry,” “there is nothing that needs to be fixed”) were coded as NA. See S1 Table for more details on the coding procedure and categories. For all other responses, response efficacy was coded as the original response to how confident the participant was that they could do something (*M* = 56.96, *SD* = 41.75).

External political efficacy. Participants indicated their agreement with two items on a scale from 1 *strongly disagree* to 5 *strongly agree*. Items measured how efficacious people are in the political process and how response the government was to their interests [30], including “People like me don't have any say about what the government does” and “I don't think public officials care much what people like me think.” Items were reverse-scored and averaged so higher scores reflect greater efficacy (*M* = 2.64, *SD* = 1.06, *r* = .50***).

Internal political efficacy. Participants indicated how much they agreed with each of three statements measuring how efficacy participants felt as political actors (i.e., “I think that I am better informed about politics and government than most people, “I consider myself to be well qualified to participate in politics”). Participants rated items on a scale from 1 *strongly disagree* to 5 *strongly agree.* The scale reflects the average of the three items with higher values indicated greater perceived efficacy (*M* = 3.67, *SD* = 0.89, α = .77).

#### Commitment to social activism.

Participants indicated how willing they would be to participate in several activism behaviors [11]. They indicated, “With regard to this presidential election, I would be willing to...” for each action from *0% willing to do this action* to *100% willing to do this action.* Participants rated 13 actions, including “volunteer for my candidate's campaign,” “post about my candidate on social media,” and “canvassing neighborhoods for my candidate.” Because we were interested in more public political activist actions, we excluded actions about talking to or educating oneself or others (e.g., “talk about the candidates with someone who agrees with me,” “educate myself on the issues”) which can more easily be done in private or more intimate settings. The scale is the sum of responses for each of the eight actions (*M* = 368.25, *SD* = 223.57).

#### Voting intention.

Participants indicated if they intended to vote by responding to the following question: “Do you intend to vote in the November 2024 election for President?” 1) yes, 2) no, 3) I don’t know, or 4) not applicable. We re-coded “I don’t know” or “not applicable” as missing data to create a dichotomized variable of voting intention that only included responses of “yes” or “no” in the analyses. The vast majority of participants intended to vote (89.61%) compared to those who did not intend to vote (4.39%) or who did not respond (6.01%).

## Results

### Analysis plan

Because the AAM predicts nonlinear relationships at various levels of efficacy, traditional interaction models may not capture these varied associations. As such, we follow best practices recommended by Mize for nonlinear interactions and present plots of predicted values from fitted models along with their corresponding marginal effects at various levels of efficacy [46]. Specifically, we fitted models with anger intensity, anger intensity squared, anger intensity cubed, efficacy, and interactions between efficacy and the anger terms as the independent variable on the outcome (i.e., commitment to social activism or intention to vote). Because the intention to vote is a binary outcome, these models leverage a generalized linear model to estimate the probability of intending to vote. Further, to understand how different variations of efficacy may interact with anger and impact prosocial political behaviors, we repeated each analysis with each of the three types of efficacy measured: response efficacy, external political efficacy, and internal political efficacy. Each model controls for the intensity of anxiety, age, gender, race (dichotomized into White and People of Color), social class (dichotomized into participants with versus without a four-year college degree), political party (dichotomized into Democrat versus Republican), and time (measured as days since the survey launch date). Full model outputs are reported in the SI.

After fitting the models, we used the “marginaleffects” package in R [48] to calculate marginal effects and second differences at four ranges of anger intensity (i.e., 0–25, 26–50, 51–75, 76–100) and three levels of efficacy (< 2: low efficacy; between 2 and 4: moderate efficacy; > 4: high efficacy). We present the marginal effects and second differences in the tables below and plot the predicted values from the model in the figure below.

## Tests of hypotheses

### Commitment to social activism

#### Response efficacy.

Results are shown in Table 1 and depicted in Fig 2. At *low levels of response efficacy*, there was no evidence for a significant association between anger and commitment to activism at low levels of anger. However, at high levels of anger, anger was positively associated with commitment to social activism. At *high levels of response efficacy*, there was no evidence for a significant relationship between anger and commitment to social activism at any range of anger intensity. Thus, as shown by the marginally significant second differences at high anger (i.e., 51–75, 76–100), anger was more *strongly* (and positively) associated with social activism at low compared to high response efficacy. See S2 Table for full model output.

**Table 1:** Average marginal effects (AMEs) and second differences between low and high response efficacy at various levels of anger on support for commitment to social activism. AME = Average Marginal Effects. Low response efficacy represents estimates at efficacy = 2 or below. High response efficacy represents estimates at efficacy = 4 and greater.

Anger Intensity	AME _Low Response Efficacy_					AME _High Response Efficacy_					Second Difference				
	*B*	*SE*	95% CI	*z*	*p*	*B*	*SE*	95% CI	*z*	*p*	*B*	*SE*	95% CI	*z*	*p*
0 - 25	55.24	43.58	[-30.17, 140.64]	1.27	.205	10.24	34.58	[-57.76, 78.25]	0.29	.768	44.99	55.30	[-63.38, 153.40]	0.81	.416
26 – 50	31.11	19.83	[-7.75, 69.96]	1.67	.117	8.75	15.32	[-21.28, 38.77]	0.57	.568	22.36	25.01	[-26.66, 71.39]	0.89	.371
51 – 75	53.09	19.59	[14.71, 91.48]	2.71	.007	4.66	15.31	[-25.36, 34.67]	0.30	.761	48.44	24.80	[-0.16, 97.04]	1.95	.051
76 - 100	117.99	52.19	[15.70, 220.29]	2.26	.024	-2.42	39.52	[-79.87, 75.03]	-0.06	.951	120.40	65.35	[-7.68, 248.50]	1.84	.065

**Fig 2:**
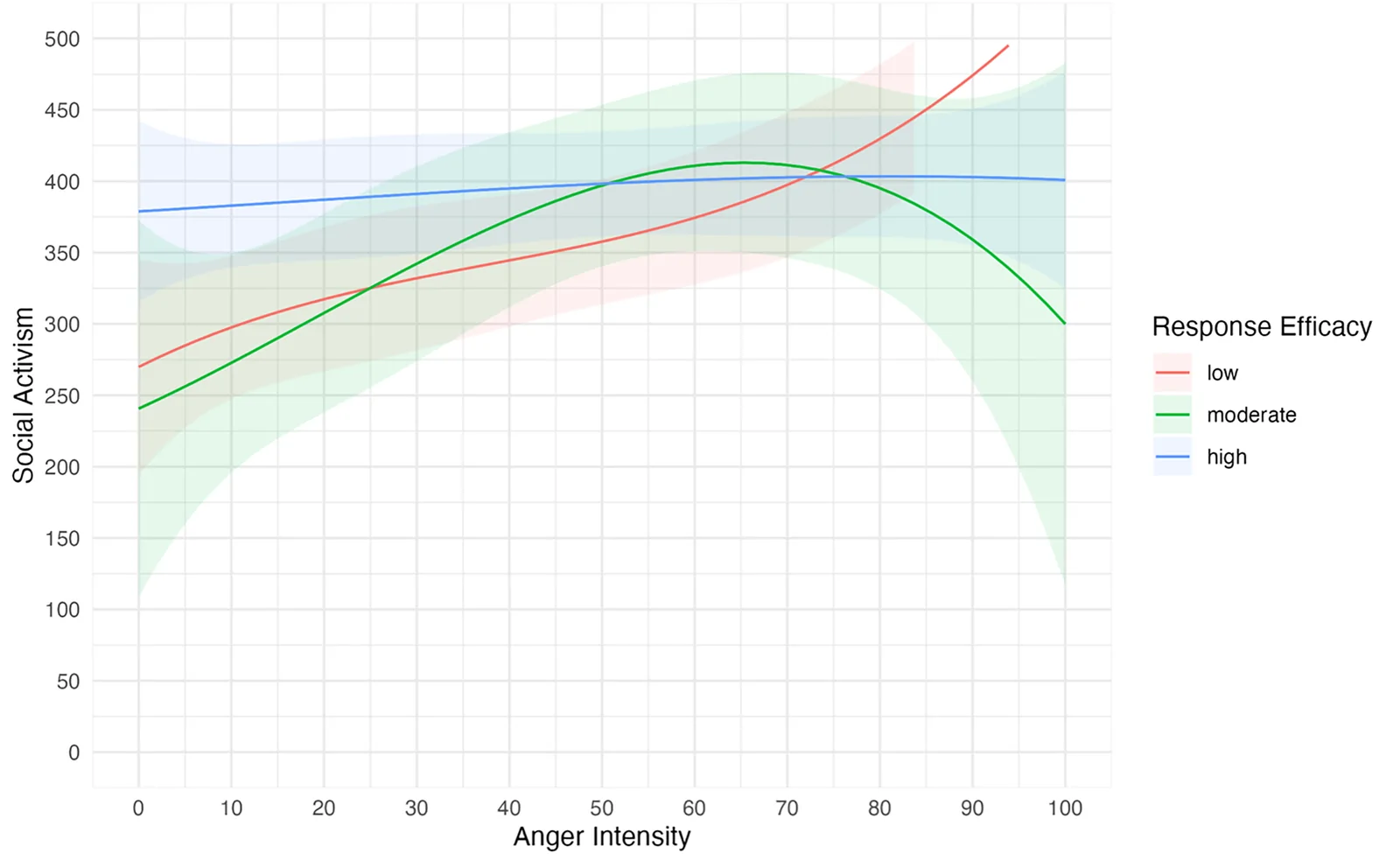
Predicted support for commitment to social activism by anger intensity and response efficacy. The social activism scale ranges from 0-800.

#### External political efficacy.

We repeated the same procedure for external political efficacy and present the results in Table 2 and Fig 3. At *low levels of external political efficacy*, there was no evidence of a significant relationship between anger and commitment to social activism at the lowest and high ranges of anger (i.e., 0–25, 76–100). At moderate ranges of anger (i.e., 26–50, 51–75), there was some evidence that anger was positively associated with commitment to social activism as shown by the significant and marginally significant associations at low external efficacy. At *high levels of external political efficacy*, there was no evidence for an association between anger and commitment to social activism at any range of anger intensity. Further, the lack of significant second differences suggests that the positive associations at low efficacy for moderate anger are not robust enough to be meaningfully different than the null associations high efficacy. See S3 Table for full model output.

**Table 2:** Average marginal effects (AMEs) and second differences between low and high external political efficacy at various levels of anger on support for commitment to social activism. AME = Average Marginal Effects. Low external efficacy represents estimates at efficacy = 2 or below. High external efficacy represents estimates at efficacy = 4 and greater.

Anger Intensity	AME _Low External Efficacy_					AME _High External Efficacy_					Second Difference				
	*B*	*SE*	95% CI	*z*	*p*	*B*	*SE*	95% CI	*z*	*p*	*B*	*SE*	95% CI	*z*	*p*
0 - 25	23.02	41.73	[-58.77, 104.80]	0.55	.581	63.79	55.62	[-45.22, 172.80]	1.15	.251	-40.77	69.51	[-177.00, 95.46]	-0.59	.558
26 – 50	34.82	17.69	[0.15, 69.48]	1.97	.049	5.26	29.66	[-52.87, 63.40]	0.18	.859	29.55	34.44	[-37.94, 97.05]	0.86	.391
51 – 75	29.46	17.43	[-4.69, 63.62]	1.69	.091	13.45	29.91	[-45.16, 72.07]	0.45	.653	16.01	34.54	[-51.69, 83.71]	0.46	.643
76 - 100	6.53	42.36	[-76.50, 89.55]	0.15	.878	83.89	87.71	[-88.03, 255.80]	0.96	.339	-77.36	97.24	[-268.00, 113.20]	-0.80	.426

**Fig 3:**
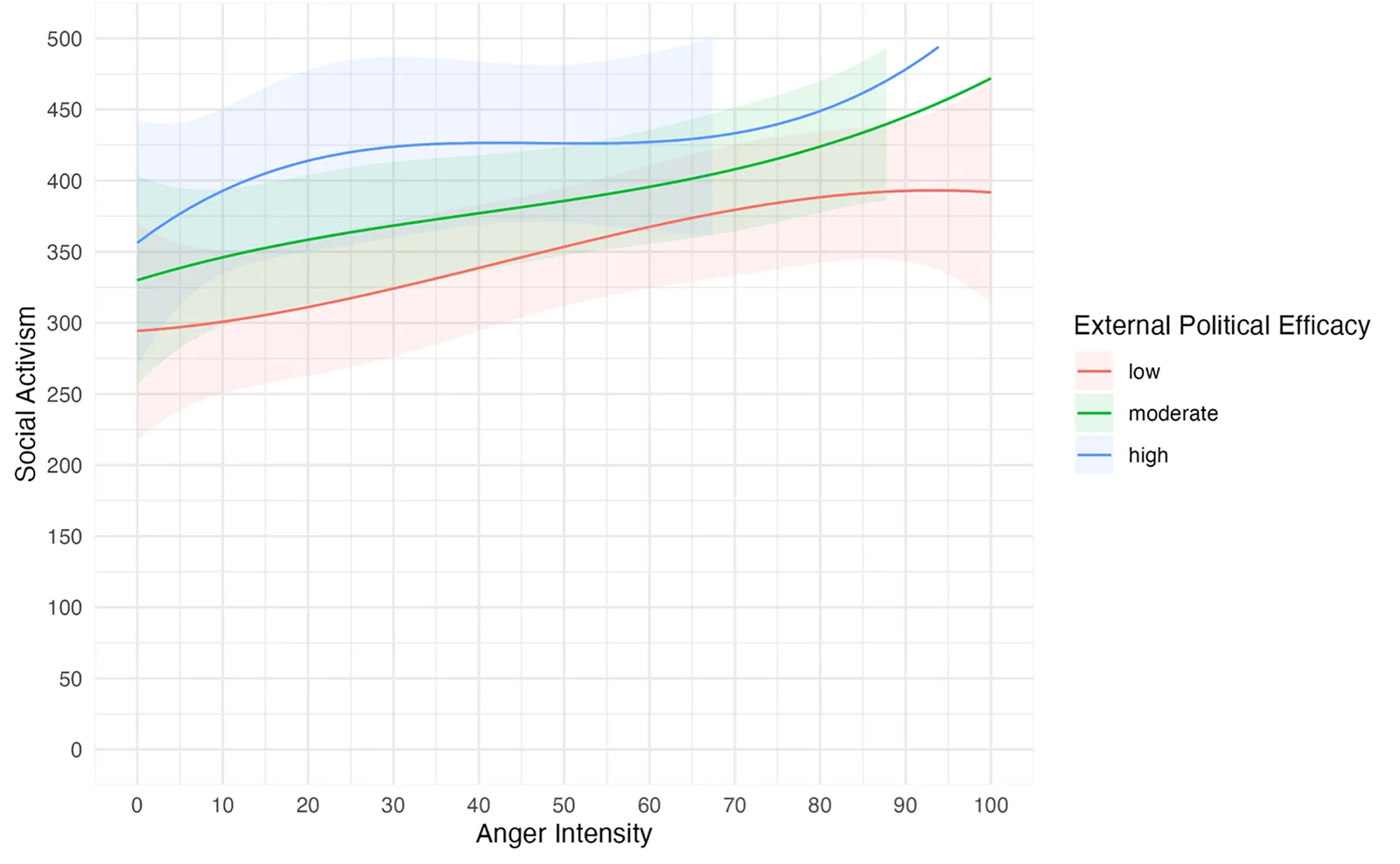
Predicted support for commitment to social activism by anger intensity and external political efficacy.

#### Internal political efficacy.

Following the same procedure, we present the results in Table 3 and Fig 4. At *low levels internal political efficacy*, the results show a significant positive relationship between anger and commitment to social activism at the lowest and highest ranges of anger (i.e., 0–25, 76–100). At moderate anger (i.e., 26–50, 51–75), there was no evidence for a relationship between anger and social activism. At *high levels of internal political efficacy*, there was no evidence for a relationship between anger and commitment to social activism at any range of anger. Thus, as suggested by the marginally significant second differences at low and high anger (i.e., 0–25, 76–100), anger had a *stronger* (and positive) association with commitment to social activism among those with low vs. high internal political efficacy. See S4 Table for full model output.

**Table 3:** Average marginal effects (AMEs) and second differences between low and high internal political efficacy at various levels of anger on support for commitment to social activism. AME = Average Marginal Effects. Low response efficacy represents estimates at efficacy = 2 or below. High response efficacy represents estimates at efficacy = 4 and greater.

Anger Intensity	AME _Low Internal Efficacy_					AME _High Internal Efficacy_					Second Difference				
	*B*	*SE*	95% CI	*z*	*p*	*B*	*SE*	95% CI	*z*	*p*	*B*	*SE*	95% CI	*z*	*p*
0 - 25	204.75	98.01	[12.61, 396.89]	2.09	.037	23.47	34.46	[-44.07, 91.02]	0.68	.496	181.30	103.50	[-21.57, 384.10]	1.75	.080
26 – 50	-22.50	45.17	[-111.03, 66.03]	-0.50	.618	12.60	15.71	[-18.18, 43.39]	0.80	.422	-35.10	47.88	[-129.00, 58.74]	-0.73	.464
51 – 75	11.42	42.95	[-72.77, 95.60]	0.27	.790	17.44	15.21	[-12.37, 47.25]	1.15	.252	-6.02	45.56	[-95.32, 83.28]	-0.13	.895
76 - 100	290.78	138.11	[20.09, 561.47]	2.11	.035	36.66	40.14	[-42.03, 115.34]	0.91	.261	254.10	144.00	[-28.07, 536.30]	1.77	.078

**Fig 4:**
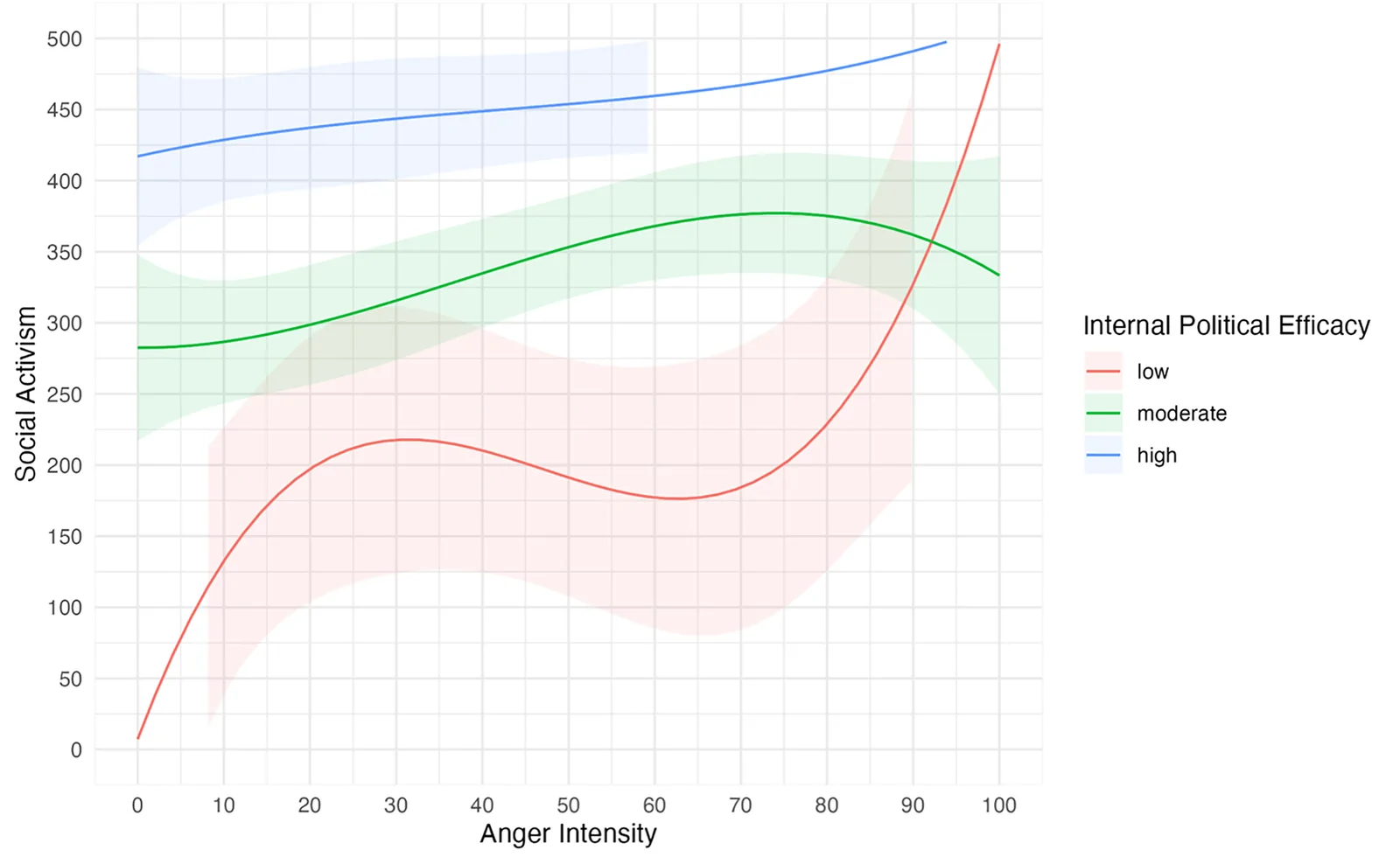
Predicted support for commitment to social activism by anger intensity and internal political efficacy.

### Voting intention

#### Response efficacy.

Following the same procedure as before, we present the results in Table 4 and Fig 5. At *low levels of response efficacy*, there was no evidence for a relationship between anger and voting intention at low and moderate anger (i.e., 0–25, 26–50, 51–75). However, at high anger intensity (i.e., 76–100), anger was negatively associated with intention to vote. At *high levels of response efficacy*, there was no evidence for significant associations between anger and voting intention at any range of anger. Thus, as indicated by the significant second difference at high anger (i.e., 76–100), anger had a *stronger* (and negative) association with voting intention at low vs. high response efficacy. See S5 Table for full model output.

**Table 4:** Average marginal effects (AMEs) and second differences between low and high response efficacy at various levels of anger on voting intention. AME = Average Marginal Effects. Low response efficacy represents estimates at efficacy = 2 or below. High response efficacy represents estimates at efficacy = 4 and greater.

Anger Intensity	AME _Low Response Efficacy_					AME _High Response Efficacy_					Second Difference				
	*B*	*SE*	95% CI	*z*	*p*	*B*	*SE*	95% CI	*z*	*p*	*B*	*SE*	95% CI	*z*	*p*
0 - 25	-0.06	0.04	[-0.14, 0.01]	-1.63	.103	0.01	0.02	[-0.04, 0.06]	0.44	.657	-0.07	0.05	[-0.16, 0.02]	-1.61	.107
26 – 50	0.03	0.03	[-0.03, 0.08]	1.01	.310	-0.01	0.01	[-0.03, 0.01]	-1.32	.186	0.04	0.03	[-0.02, 0.09]	1.35	.178
51 – 75	0.01	0.02	[-0.03, 0.04]	0.39	.699	-0.01	0.02	[-0.04, 0.02]	-0.85	.395	0.02	0.02	[-0.03, 0.07]	0.85	.397
76 - 100	-0.22	0.11	[-0.44, -0.003]	-1.98	.047	0.02	0.02	[-0.02, 0.07]	0.99	.321	-0.25	0.11	[-0.47, -0.02]	-2.15	.032

**Fig 5:**
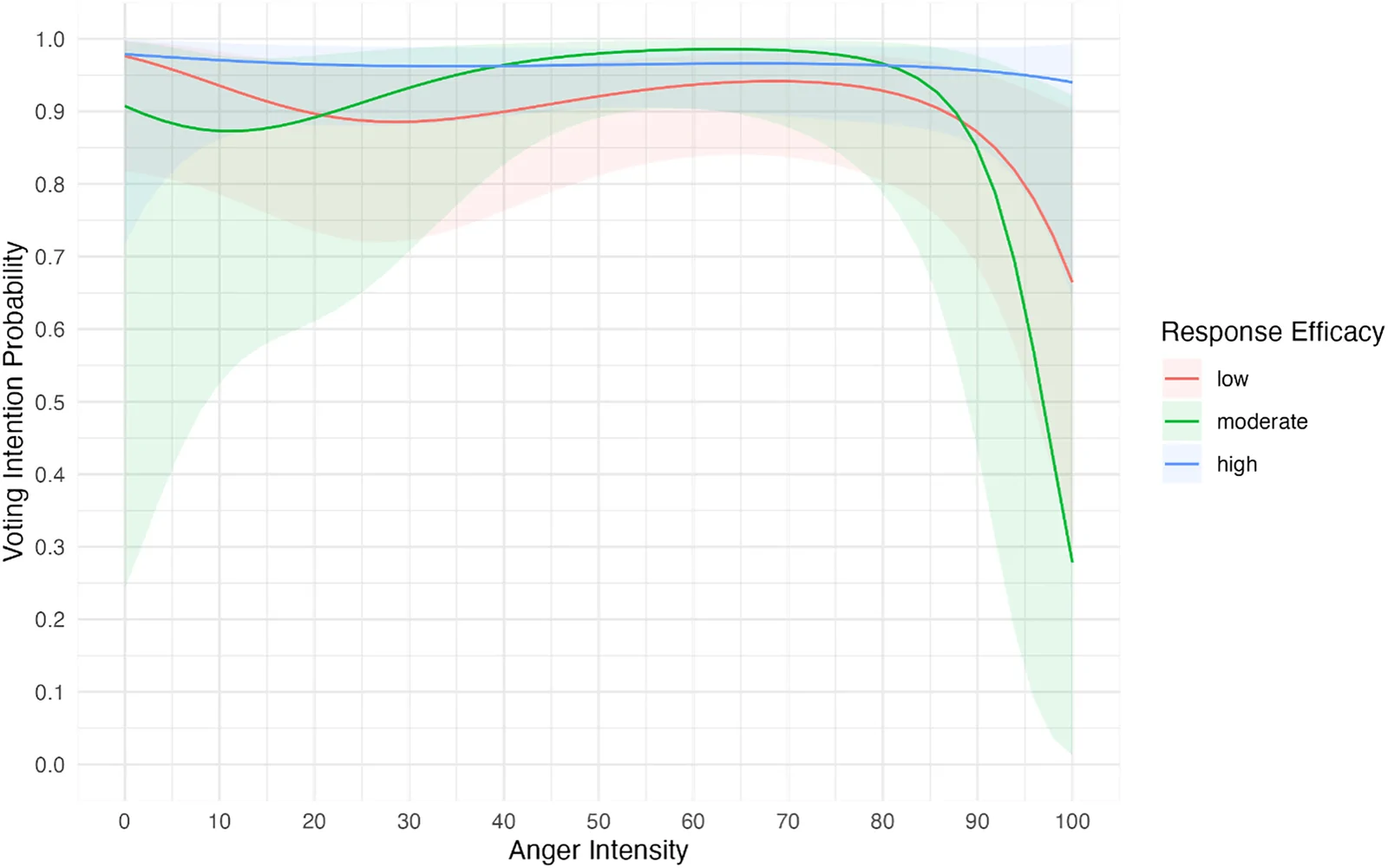
Predicted support for voting intention by anger intensity and response efficacy.

#### External political efficacy.

When fitting the model using external political efficacy, we discovered that there was nearly zero variance in the outcome among participants with the highest levels of efficacy. Specifically, all but one of the participants who reported high external political efficacy indicated that they intended to vote in the upcoming election (n = 165). The lack of variance in the outcome at high levels of efficacy made analyses of marginal effects and second differences in this condition inappropriate given there was variation in the outcome at three of the four ranges of anger intensity. As such, we adjusted the model to only include participants in the low and moderate efficacy groups and present the marginal effects at low external political efficacy in Table 5 and plot the predict values in Fig 6. We did not compare second differences between low and high efficacy, given the absences of meaningful variation in the outcome at high efficacy. The results do not show significant associations between anger intensity and voting intention at low efficacy at any range of anger. See S6 Table for full model output.

**Table 5:** Average marginal effects (AMEs) at low external political efficacy at various levels of anger on voting intention. AME = Average Marginal Effects. Low response efficacy represents estimates at efficacy = 2 or below. High response efficacy represents estimates at efficacy = 4 and greater.

Anger Intensity	AME _Low External Efficacy_					AME _High External Efficacy_					Second Difference				
	*B*	*SE*	95% CI	*z*	*p*	*B*	*SE*	95% CI	*z*	*p*	*B*	*SE*	95% CI	*z*	*p*
0 - 25	0.0003	0.05	[-0.09, 0.09]	0.007	.994	--	--	--	--	--	--	--	--	--	--
26 – 50	0.02	0.02	[-0.02, 0.05]	0.97	.334	--	--	--	--	--	--	--	--	--	--
51 – 75	-0.002	0.01	[-0.03, 0.02]	-0.19	.848	--	--	--	--	--	--	--	--	--	--
76 - 100	-0.10	0.06	[-0.23, 0.03]	-1.57	.117	--	--	--	--	--	--	--	--	--	--

**Fig 6:**
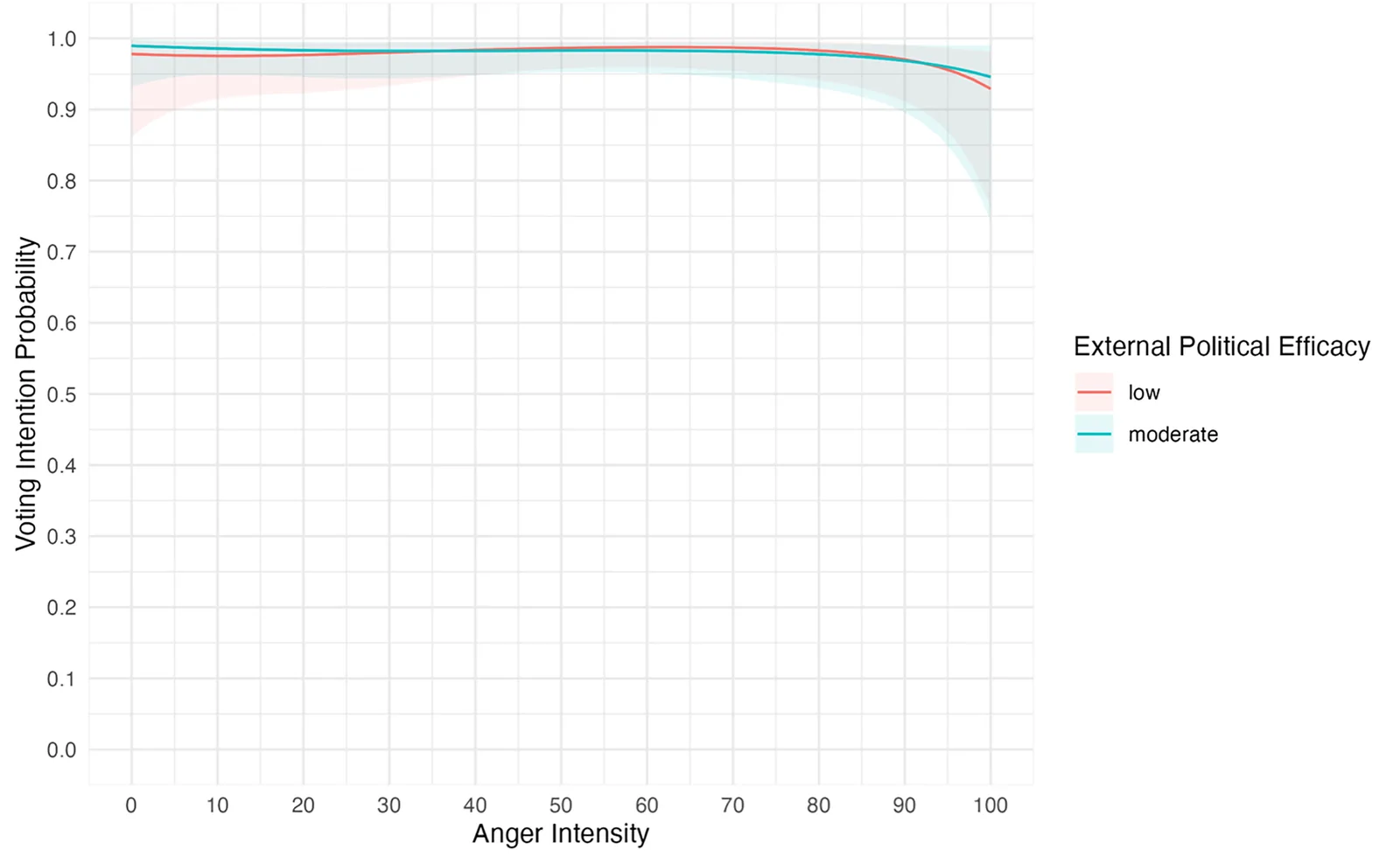
Predicted support for voting intention by anger intensity and external political efficacy.

#### Internal political efficacy.

Following the same procedure as before, we present the results in Table 6 and Fig 7. The analyses did not reveal significant associations between anger and voting intention at low or high internal political efficacy at any range of anger. Further, there was no evidence for second differences between low and high internal political efficacy. See S7 Table for full model output.

**Table 6:** Average marginal effects (AMEs) and second differences between low and high internal political efficacy at various levels of anger on voting intention. AME = Average Marginal Effects. Low internal efficacy represents estimates at efficacy = 2 or below. High internal efficacy represents estimates at efficacy = 4 and greater.

Anger Intensity	AME _Low Internal Efficacy_					AME _High Internal Efficacy_					Second Difference				
	*B*	*SE*	95% CI	*z*	*p*	*B*	*SE*	95% CI	*z*	*p*	*B*	*SE*	95% CI	*z*	*p*
0 - 25	-0.12	0.19	[-0.50, 0.25]	-0.64	.521	-0.001	0.01	[-0.02, 0.02]	-0.11	.910	-0.12	0.19	[-0.50, 0.25]	-0.63	.526
26 – 50	0.11	0.10	[-0.09, 0.31]	1.08	.278	-0.005	0.01	[-0.02, 0.01]	-0.78	.437	0.12	0.10	[-0.09, 0.32]	1.13	.259
51 – 75	0.02	0.07	[-0.11, 0.15]	0.34	.733	-0.01	0.01	[-0.03, 0.02]	-0.52	.602	0.03	0.07	[-0.10, 0.16]	0.43	.670
76 - 100	-0.43	0.33	[-1.08, 0.22]	-1.29	.198	0.002	0.03	[-0.05, 0.06]	0.07	.942	-0.43	0.33	[-1.08, 0.22]	-1.29	.197

**Fig 7:**
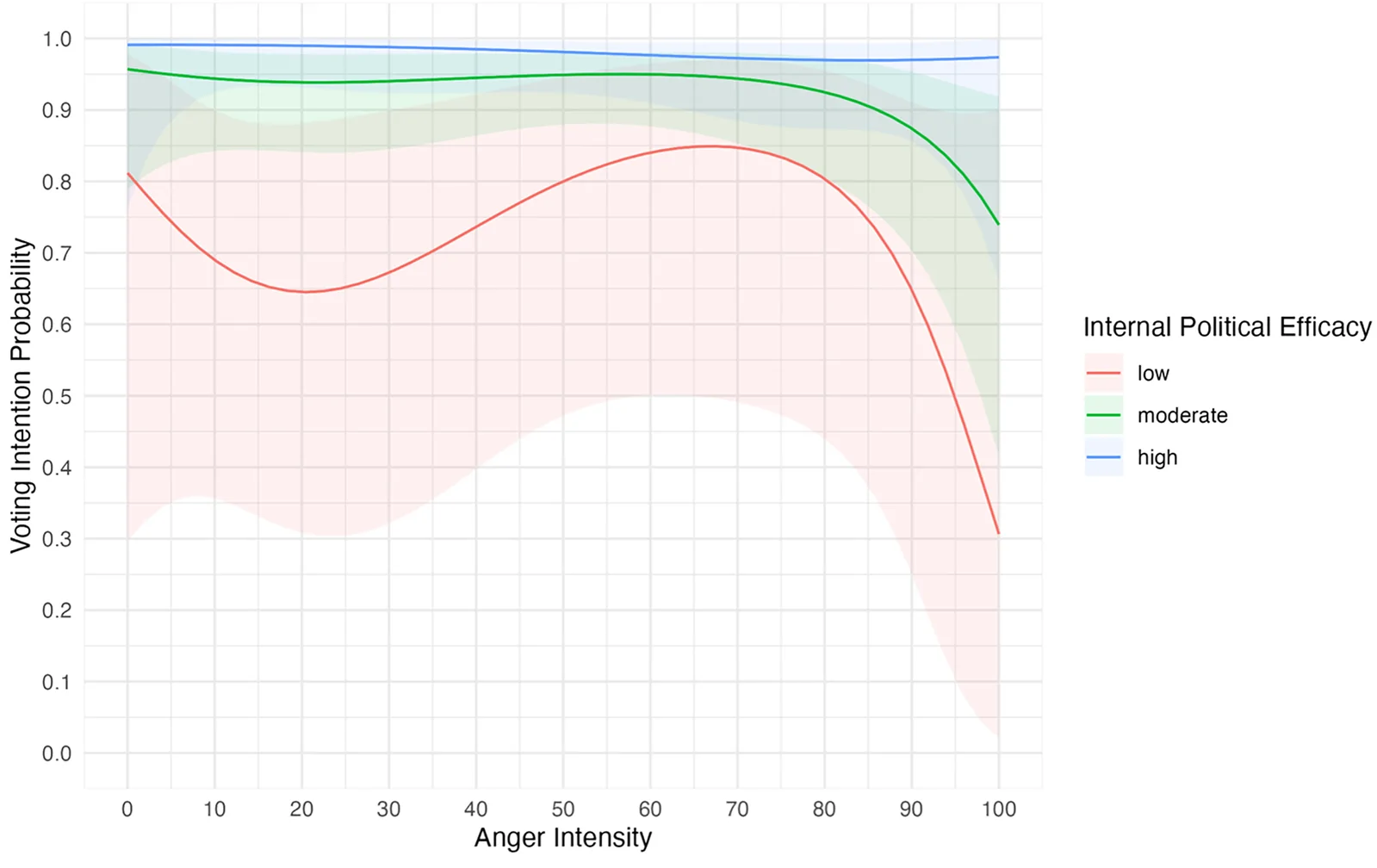
Predicted support for voting intention by anger intensity and internal political efficacy.

## Discussion

This study sought to examine the relationship between anger and prosocial outcomes during the weeks preceding a contentious and polarizing presidential election. Informed by the Anger Activism Model [7,15], we predicted that the relationship between anger and outcomes is conditional: When efficacy is high, anger will be positively and linearly associated with political social activism and the probability of voting, and when efficacy is weak, the relationship should be nonmonotonic [11,14,25,26].

Using aggregated (rolling) cross-sectional data from the five weeks immediately prior to the election, we examined anger’s relationship to prosocial political outcomes—commitment to social activism and voting intention—and whether multiple forms of efficacy moderate these relationships [7,15]. The evidence provides a nuanced picture that partly confirms expectations yet runs counter to predictions in key ways.

First, the relation between anger and activism was contingent on efficacy type and level. When response efficacy was low, increases in anger were associated with increasing activism; particularly at higher anger intensities (i.e., when anger was higher than 50 on a 101-point scale). At high response efficacy, by contrast, anger showed no detectable association with activism—anger was irrelevant for those who believe there is a solution for their angering problem. Interestingly, the data reveals that at high levels of response efficacy, there was little variance in social activism intentions; scores were high across the board. But, for low response efficacy and weak anger, activism scores were very low and steadily increased.

For external political efficacy, we observed no reliable second differences or marginal effects across anger intensities. Examining the plots though, patterns resembled those for response efficacy: little variance in the dependent variable for high internal efficacy. And, when internal efficacy is weak the plot suggests a positive linear relationship with outcomes. Regarding internal political efficacy, we again see little variance in the outcomes for those with high scores. But, when internal efficacy is low we see a quadratic relationship.

Second, for voting intention, anger again interacted with efficacy, with the clearest pattern under internal efficacy: at low internal efficacy, very high anger related to lower likelihood of intending to vote—consistent with the AAM. However, estimates near zero emerged at high internal efficacy; simply because there was nearly zero variance in voting intent. For response efficacy, the pattern is similar to that of internal efficacy: low-efficacy respondents showed small negative or null associations at very high anger, with weak evidence of positive associations at moderate anger ranges. External efficacy yielded few interpretable patterns.

These data provide important insights regarding the predictive validity of the AAM in the midst of an election. First, efficacy of all kinds is strongly related to voting behaviors and political engagement (social activism). When efficacy was at its highest, there was nearly zero variance in intentions for this sample. But, moderate and low efficacy moderates the relationship between anger and political behaviors (i.e., activism and voting). When anger is very low and efficacy is low we find an unengaged audience—consistent with the AAM. In these conditions, as anger creeps up so do intentions, which is also consistent with the AAM.

Yet, the findings for low/moderate efficacy and high anger are different for the two outcomes we measured. Activism is higher at high levels of anger even when efficacy is low. These individuals appear to be willing to engage in the political process by volunteering and assisting campaigns. That said, high anger and low/moderate efficacy are associated with lower voting intentions. It is possible that at high anger and low efficacy people perceive that their vote does not matter. Yet, still see value in contributing to their candidate’s success.

### Theoretical implications

In summary, the AAM anticipates two broad findings: (a) when efficacy is strong, anger should show a positive, approximately linear relation with prosocial outcomes; (b) when efficacy is weak, the relation should be nonmonotonic, with moderate anger sometimes associated with action but very high anger associated with lower engagement [7,15]. Our field data align partly with these expectations.

There is partial alignment with predictions under weak efficacy. For internal efficacy, we observe the predicted nonmonotonicity across outcomes: extremely high anger at low efficacy relates to reduced voting intention (consistent with overwhelm/reactance), yet increased activism (consistent with approach-oriented outlet seeking) [20,44]. For response efficacy, high anger at low efficacy is associated with greater activism, again implying an approach motive when a pathway (even if not fully effective) is salient to the individual [40].

There is deviation from linearity under strong efficacy. Contrary to our prediction, we do not observe robust, linear relationships between anger and outcomes at high efficacy—estimates are small and statistically indistinguishable from zero across most ranges. Two nonexclusive explanations are plausible and consistent with these data: There could be ceiling/threshold dynamics in naturalistic contexts. In a presidential election, high-efficacy citizens may already be at or near an activation set-point (e.g., stable turnout intentions, routine civic behaviors); additional anger is associated with little additional engagement [37,38]. It could also be that the form of efficacy that matters may be proximal to the behavior (e.g., internal confidence to act or response beliefs about tactics) rather than system-level trust (external efficacy) or a generalized “high” efficacy state. If proximal efficacy is already high, anger’s incremental association with participation may be muted [14,25,26]. And we should consider that efficacy is multidimensional—and not interchangeable. The weak or null moderating role of external efficacy suggests that system-level beliefs about government responsiveness are less proximal to short-run mobilization than internal (can I/we do this?) or response (will this tactic work?) efficacy. Because we measured response, internal political, and external political efficacy in the same sample and modeled each in parallel, this is a direct rather than an across-study comparison, which the AAM literature has not previously provided. It offers an extension to the AAM: efficacy proximity (to the contemplated action) may condition whether anger is associated with participation [7,15].

Our results complement laboratory evidence showing that anger is associated with both approach-oriented engagement and reduced deliberative, cooperative behavior depending on context, intensity, and perceived control [5,21,42,43]. In this naturalistic environment, very high anger appears especially unstable in its associations: it is related to greater activism among those who perceive personal or tactical capacity (internal/response efficacy), but to lower voting intention among those who doubt their own political competence. Thus, the same emotional state is associated with engagement in one behavior and disengagement from another, depending on which efficacy belief is salient and which behavior is at stake [20,37].

There are critical theoretical implications. First, the AAM should be refined to consider efficacy proximity. Our results suggest adding a proximity principle: the moderating potency of efficacy increases as it becomes more behavior-proximal (internal/response > external) [14,15]. Second, in some conditions there may be bounded linearity at high efficacy. In highly mobilizing environments (e.g., a national election), baseline engagement among high-efficacy individuals may attenuate anger’s marginal associations, producing flat slopes even when theory predicts linearity; AAM predictions may therefore be state-dependent, varying with contextual mobilization levels [36]. Third, there may be outcome specificity. Associations diverged for activism versus voting, indicating that “prosocial political action” is not unitary and should not be treated as a single construct. Theories should model behavior-specific pathways, recognizing that the cognitive and logistical demands of voting differ from those of activism (e.g., threshold, identity signaling, immediacy of feedback) [40].

### Practical implications

Additionally, there are practical implications for communicators and organizers. First, it is advisable to pair anger with proximal efficacy cues. Appeals that highlight concrete, doable actions (response efficacy) and people’s capacity to execute them (internal efficacy) are more likely to channel anger into activism [15,25,49,50]. Second, avoid maximal anger in low-efficacy audiences for turnout goals. When the objective is voting, extreme anger without efficacy scaffolding risks demobilization among the least internally efficacious [20,44]. Third, segment by efficacy profile [14]. Diagnostics that identify internal vs. external vs. response efficacy deficits can guide tailored messaging (skill-building and planning prompts for low-internal; tactic effectiveness evidence for low-response; institutional responsiveness cues for low-external). Finally, design pathways for resolution and empowerment, not just emotions. Anger can open the door, but clear, credible pathways must be visible immediately; otherwise, the energy may dissipate into cynicism or nonvoting [5,21].

### Limitations and future directions

There are strengths and limitations to this study. Strengths include (a) a field test of emotion–efficacy dynamics during a major national election, (b) a rolling cross-section design that samples across weeks and news cycles, (c) simultaneous assessment of three efficacy constructs, and (d) modeling of nonlinear interactions using marginal effects and second differences [46]. Several limitations qualify interpretation. First, although we leverage timing and controls, the design remains observational; causal claims are not warranted [51]. That said, the current study is an extension of (and not a substitution for) experimental AAM studies where anger was manipulated [11,15]. The findings reported here, in the main, map on to the predictions of the AAM for internal and external political efficacy, providing some confidence in these correlational findings. Relatedly, our outcomes were limited to legal, nonviolent forms of participation. We did not measure non-normative or antisystem outcomes, such as support for political violence or anti-democratic practices, and these data therefore cannot speak to whether anger and efficacy are associated with those behaviors in the same way. Second, anger and efficacy were self-reported; common-method variance and social desirability are possible [52]. Third, the anger composite included angry and mad; while conceptually coherent, different facets might differentially predict outcomes [18,19]. Also, the anger measure asked people to refer to the “2024 election season”. As a result, respondents referred to various specific targets of their anger, and not one specific stimuli. It is also the case that some respondents could be the type of person who feels angry about an array of events (related and unrelated to politics); since we did not measure and control for trait anger, we suggest this variable be accounted for in future studies. We recommend that future field studies disaggregate targets of anger on outcomes. Fourth, voting was measured as intention (or self-reported behavior for post-Election respondents) and dichotomized after excluding “don’t know”/NA, which may attenuate nuance in ambivalence. Fifth, the sample, though quota-balanced to approximate national demographics, was recruited from an online panel and may differ from the full electorate on unmeasured characteristics. Sixth, results are embedded in the 2024 U.S. election context; generalization to lower-salience elections or non-U.S. settings should be made cautiously [36]. Finally, this study was not preregistered. We were extending the AAM in two ways: applying it to organically occurring (rather than experimentally manipulated) anger during an active presidential campaign and incorporating two new forms of efficacy: internal and external political efficacy. Given these extensions, determining the appropriate functional form for anger, including the possibility of nonlinear effects under conditions of low efficacy, required development. We approached this in good faith through extensive theoretical refinement and by consulting established best practices for specifying and interpreting nonlinear interaction models (e.g., Mize, 2019). This process was intended to ensure that our analytic decisions were grounded in theory and methodological standards rather than ad hoc exploration. Even so, we acknowledge that preregistered replications will be essential for future work in this area.

We would like to note some future directions that scholars might take. Regarding experimental and natural experiments, we suggest combining exogenous anger inductions with efficacy manipulations in ecologically valid tasks (e.g., ballot request completion, donation to real organizations) to isolate mechanisms and test the efficacy proximity refinement [15,25,26]. Also, regarding behavioral outcomes, we suggest linking survey responses to administrative turnout records and digital trace measures of activism (e.g., verified petition signatures) to reduce reporting bias. Likewise, use of panel or experience-sampling designs to model how anger and efficacy co-evolve across events could allow for greater distinction between integral and incidental anger in real time [42,43]. Additionally, other moderators should be considered such as issue ownership, partisan identity strength, certainty/appraisal patterns, and moral conviction that may amplify or dampen efficacy’s conditioning role [5,18,20].

### Summary

In summary, this study shows that anger’s associations with political outcomes depend on which form of efficacy citizens bring to bear and on how intense their anger becomes. In the run-up to a high-stakes presidential election, very high anger paired with low internal efficacy was associated with lower voting intention, yet with greater activism when response or internal efficacy was salient. We find little evidence that anger is associated with additional mobilization among high-efficacy citizens in this context, suggesting bounded returns of anger when baseline activation is already high. Together, these findings refine the AAM by testing it in the field rather than the laboratory, by distinguishing among three forms of efficacy, and by highlighting efficacy proximity, outcome specificity, and contextual ceilings, and they offer a practical lesson: anger needs a ladder. Without credible steps people believe they can climb, its energy may burn hot but not helpfully. With the right rungs in place, anger can be a civic resource rather than a civic risk [7,15,40].

## Supporting information

S1 Table Open-ended data coding scheme. (DOCX)

S2 Table Full model output for commitment to social activism and response efficacy. (DOCX)

S3 Table Full model output for commitment to social activism and external political efficacy. (DOCX)

S4 Table Full model output for commitment to social activism and internal political efficacy. (DOCX)

S5 Table Full model output for voting intention and response efficacy. (DOCX)

S6 Table Full model output for voting intention and external political efficacy. (DOCX)

S7 Table Full model output for voting intention and internal political efficacy. (DOCX)
