# Supplementary material for: From outrage to action: Efficacy moderates the relationship between anger and prosocial political outcomes
Source: PLoS One. 2026 Oct 14;21(10):e0358939. doi: 10.1371/journal.pone.0358939 (PMC13667767; doi:10.1371/journal.pone.0358939)
Supplement: Supplementary file 1 [file pone.0358939.s001.docx]

**S1 Table.** **Open-ended data coding scheme**

| Code | Definition / Notes | Examples | Kappa | Incorporated into Response Efficacy |
| --- | --- | --- | --- | --- |
| Not angry | Any response that says nothing can be done because they are not angry (so therefore there is no problem to address). Do not double code with “nothing” or “NA.” | *“not applicable I was not angry”, “No and because I am happy and proud of her,” “[NA] I am not angry at the election but I am due to the stupid query,” “[NA] not applicable I was not angry,’ “[NA] no anger”* | .72 | Treated as NA for this analysis |
| Problem solved or No Problem | Responses that reference or imply that the problem already has been solved and actions in the past solved this problem. This also includes statements that there is no problem to be solved. | *“We addressed it by voting Trump,” “TRUMP WON:)” “The is nothing that needs to be fixed”* | .67 | Treated as NA for this analysis |
| Not applicable | If response says “not applicable” then code as “NA.” If they specifically say it’s not applicable because they are not angry about the election, then code as “nothing b/c not angry” (and do not code here). | *“[NA] not applicable”* | 1 | Treated as NA for this analysis |
| Nothing can be done | Any response that says nothing can be done. Includes responses that they don’t know or are unsure what can be done. Do not double code with “nothing because angry” (i.e., code as one or the other). | *“No”, “Nothing she is winn,” “not sure,” “don’t know”* | .92 | Coded as “0” for response efficacy because the sentiment is that nothing can be done (i.e., low response efficacy) |
| *Note*. Two independent raters coded each open-ended response to the prompt: “Is there anything specific that can be done to address this problem [*primary target of their anger piped in*]?”  The coders rated the response on a number of dimensions (e.g., if the response mentioned using voting power as a way to address the problem). For each round of coding, the two raters coded 10% of the full corpus. We calculated inter-rater reliability by using Cohen’s kappa. Discrepancies were resolved by the two raters and a lead member of the research team. After six rounds of coding, every category had a kappa above 0.65 (average kappa across the categories = .88), the rest of the corpus was split among the two raters to each code. Kappas are reported from round 6 of coding, which was the final round before splitting the remainder of the corpus. Because “not angry” was not mentioned in round 6 of coding, the average kappa across rounds 1-5 is reported. | | | | |
