# Supplementary material for: From outrage to action: Efficacy moderates the relationship between anger and prosocial political outcomes
Source: PLoS One. 2026 Oct 14;21(10):e0358939. doi: 10.1371/journal.pone.0358939 (PMC13667767; doi:10.1371/journal.pone.0358939)
Supplement: Supplementary file 2 [file pone.0358939.s002.docx]

**S2 Table. Full model output for commitment to social activism and response efficacy**

| **Model Parameter** | **B** | **SE** | ***t*** | ***p*** |
| --- | --- | --- | --- | --- |
| Response Efficacy (linear) | 21.66 | 11.10 | 1.95 | .051 |
| Response Efficacy (quadratic) | 21.16 | 16.43 | 1.29 | .198 |
| Anger | 1156.42 | 289.75 | 3.99 | < .001 |
| Anger squared | -321.01 | 281.30 | -1.14 | .254 |
| Anger cubed | -22.85 | 272.97 | -0.08 | .933 |
| Anxiety intensity | 0.49 | 0.27 | 1.82 | .070 |
| Age | -2.25 | 0.47 | -4.78 | < .001 |
| Gender (non-binary) | 104.97 | 153.26 | 0.69 | .494 |
| Gender (woman) | -39.17 | 14.15 | -2.77 | .006 |
| Race (White) | -45.64 | 15.93 | -2.87 | .004 |
| Social Class (lower) | -14.35 | 14.64 | -0.98 | .327 |
| Political Party (Republican) | -32.59 | 14.43 | -2.26 | .024 |
| Time | -0.30 | 0.72 | -0.42 | .676 |
| Response Efficacy (linear) × Anger | -1217.42 | 350.35 | -3.48 | < .001 |
| Response Efficacy (quadratic) × Anger | -117.00 | 586.36 | -0.20 | .842 |
| Response Efficacy (linear) × Anger squared | -365.82 | 349.95 | -1.05 | .296 |
| Response Efficacy (quadratic) × Anger squared | 1207.29 | 593.61 | 2.03 | .042 |
| Response Efficacy (linear) × Anger cubed | -254.99 | 353.90 | -0.72 | .471 |
| Response Efficacy (quadratic) × Anger cubed | 440.21 | 566.42 | 0.78 | .437 |
| *Note*. Efficacy is an ordinal variable (low, moderate, and high). Gender is coded as men = 0. Race is coded as Participants of Color = 0. Social class is coded as higher social class contexts = 0. Political party is coded as Democrat = 0. | | | | |
