# Supplementary material for: From outrage to action: Efficacy moderates the relationship between anger and prosocial political outcomes
Source: PLoS One. 2026 Oct 14;21(10):e0358939. doi: 10.1371/journal.pone.0358939 (PMC13667767; doi:10.1371/journal.pone.0358939)
Supplement: Supplementary file 3 [file pone.0358939.s003.docx]

**S3 Table. Full model output for commitment to social activism and external political efficacy**

| **Model Parameter** | **B** | **SE** | ***t*** | ***p*** |
| --- | --- | --- | --- | --- |
| External Political Efficacy (linear) | 55.71 | 15.09 | 3.69 | < .001 |
| External Political Efficacy (quadratic) | 0.03 | 12.26 | 0.003 | .998 |
| Anger | 1064.06 | 273.26 | 3.89 | < .001 |
| Anger squared | 49.55 | 261.64 | 0.19 | .850 |
| Anger cubed | 165.10 | 257.26 | 0.64 | .521 |
| Anxiety intensity | 0.62 | 0.27 | 2.29 | .022 |
| Age | -2.17 | 0.47 | -4.59 | < .001 |
| Gender (non-binary) | 74.44 | 154.50 | 0.48 | .630 |
| Gender (woman) | -41.25 | 14.22 | -2.90 | .004 |
| Race (White) | -43.12 | 15.91 | -2.71 | .007 |
| Social Class (lower) | -14.70 | 14.69 | -1.00 | .317 |
| Political Party (Republican) | -24.59 | 14.67 | -1.68 | .094 |
| Time | -0.34 | 0.72 | -0.47 | .636 |
| External Political Efficacy (linear) × Anger | -52.23 | 490.86 | -0.11 | .915 |
| External Political Efficacy (quadratic) × Anger | -68.21 | 408.89 | -0.17 | .868 |
| External Political Efficacy (linear) × Anger squared | 136.71 | 491.13 | 0.28 | .781 |
| External Political Efficacy (quadratic) × Anger squared | -117.79 | 411.99 | -0.29 | .775 |
| External Political Efficacy (linear) × Anger cubed | 443.52 | 483.42 | 0.92 | .359 |
| External Political Efficacy (quadratic) × Anger cubed | 25.35 | 401.20 | 0.06 | .950 |
| *Note*. Efficacy is an ordinal variable (low, moderate, and high). Gender is coded as men = 0. Race is coded as Participants of Color = 0. Social class is coded as higher social class contexts = 0. Political party is coded as Democrat = 0. | | | | |
