# Supplementary material for: From outrage to action: Efficacy moderates the relationship between anger and prosocial political outcomes
Source: PLoS One. 2026 Oct 14;21(10):e0358939. doi: 10.1371/journal.pone.0358939 (PMC13667767; doi:10.1371/journal.pone.0358939)
Supplement: Supplementary file 4 [file pone.0358939.s004.docx]

**S4 Table. Full model output for commitment to social activism and internal political efficacy**

| **Model Parameter** | **B** | **SE** | ***t*** | ***p*** |
| --- | --- | --- | --- | --- |
| Internal Political Efficacy (linear) | 182.27 | 21.10 | 8.64 | < .001 |
| Internal Political Efficacy (quadratic) | -9.46 | 14.33 | -0.66 | .509 |
| Anger | 1315.57 | 345.64 | 3.81 | < .001 |
| Anger squared | 22.46 | 357.14 | 0.06 | .950 |
| Anger cubed | 567.44 | 345.80 | 1.64 | .101 |
| Anxiety intensity | 0.58 | 0.26 | 2.26 | .024 |
| Age | -2.17 | 0.45 | -4.86 | < .001 |
| Gender (non-binary) | 164.69 | 147.46 | 1.12 | .264 |
| Gender (woman) | -22.01 | 13.58 | -1.62 | .105 |
| Race (White) | -50.19 | 15.14 | -3.32 | < .001 |
| Social Class (lower) | 20.37 | 14.34 | 1.42 | .156 |
| Political Party (Republican) | -36.83 | 13.68 | -2.69 | .007 |
| Time | -0.12 | 0.68 | -0.17 | .862 |
| Internal Political Efficacy (linear) × Anger | -1123.49 | 678.79 | -1.66 | .098 |
| Internal Political Efficacy (quadratic) × Anger | 460.81 | 471.56 | 0.98 | .329 |
| Internal Political Efficacy (linear) × Anger squared | -236.95 | 726.84 | -0.33 | .744 |
| Internal Political Efficacy (quadratic) × Anger squared | 570.98 | 494.18 | 1.16 | .248 |
| Internal Political Efficacy (linear) × Anger cubed | -1283.74 | 696.89 | -1.84 | .066 |
| Internal Political Efficacy (quadratic) × Anger cubed | 1110.37 | 478.54 | 2.32 | .021 |
| *Note*. Efficacy is an ordinal variable (low, moderate, and high). Gender is coded as men = 0. Race is coded as Participants of Color = 0. Social class is coded as higher social class contexts = 0. Political party is coded as Democrat = 0. | | | | |
