# Supplementary material for: From outrage to action: Efficacy moderates the relationship between anger and prosocial political outcomes
Source: PLoS One. 2026 Oct 14;21(10):e0358939. doi: 10.1371/journal.pone.0358939 (PMC13667767; doi:10.1371/journal.pone.0358939)
Supplement: Supplementary file 5 [file pone.0358939.s005.docx]

**S5 Table. Full model output for voting intention and response efficacy**

| **Model Parameter** | **B** | **SE** | ***z*** | ***p*** |
| --- | --- | --- | --- | --- |
| Response Efficacy (linear) | 0.60 | 0.30 | 1.99 | .047 |
| Response Efficacy (quadratic) | -0.25 | 0.53 | -0.47 | .638 |
| Anger | -2.33 | 6.75 | -0.34 | .730 |
| Anger squared | -11.94 | 6.65 | -1.80 | .073 |
| Anger cubed | -5.41 | 6.87 | -0.79 | .431 |
| Anxiety intensity | 0.005 | 0.007 | 0.77 | .442 |
| Age | 0.03 | 0.01 | 2.61 | .009 |
| Gender (non-binary) | 9.08 | 882.74 | 0.01 | .992 |
| Gender (woman) | -0.70 | 0.38 | -1.83 | .067 |
| Race (White) | -0.72 | 0.41 | -1.74 | .082 |
| Social Class (lower) | -0.90 | 0.46 | -1.96 | .050 |
| Political Party (Republican) | 1.29 | 0.42 | 3.09 | .002 |
| Time | -0.02 | 0.02 | -1.19 | .235 |
| Response Efficacy (linear) × Anger | 1.50 | 9.37 | 0.16 | .873 |
| Response Efficacy (quadratic) × Anger | -14.03 | 13.08 | -1.07 | .283 |
| Response Efficacy (linear) × Anger squared | 3.98 | 9.32 | 0.43 | .669 |
| Response Efficacy (quadratic) × Anger squared | 26.51 | 13.45 | 1.97 | .049 |
| Response Efficacy (linear) × Anger cubed | 18.86 | 9.49 | 1.99 | .047 |
| Response Efficacy (quadratic) × Anger cubed | 7.04 | 13.94 | 0.50 | .614 |
| *Note*. Efficacy is an ordinal variable (low, moderate, and high). Logistic regression coefficients are reported. Gender is coded as men = 0. Race is coded as Participants of Color = 0. Social class is coded as higher social class contexts = 0. Political party is coded as Democrat = 0. | | | | |

**S6 Table. Full model output for voting intention and external political efficacy**

| **Model Parameter** | **B** | **SE** | ***z*** | ***p*** |
| --- | --- | --- | --- | --- |
| External Political Efficacy (low vs. moderate) | 0.008 | 0.27 | 0.03 | .977 |
| Anger | -4.57 | 5.60 | -0.82 | .415 |
| Anger squared | -6.63 | 5.11 | -1.30 | .194 |
| Anger cubed | -6.01 | 5.13 | -1.17 | .241 |
| Anxiety intensity | 0.007 | 0.007 | 1.05 | .292 |
| Age | 0.03 | 0.01 | 2.41 | .016 |
| Gender (non-binary) | 11.22 | 882.74 | 0.01 | .990 |
| Gender (woman) | -0.81 | 0.38 | -2.14 | .032 |
| Race (White) | -0.59 | 0.40 | -1.47 | .141 |
| Social Class (lower) | -1.03 | 0.47 | -2.20 | .028 |
| Political Party (Republican) | 1.28 | 0.41 | 3.11 | .002 |
| Time | -0.02 | 0.02 | -1.22 | .223 |
| External Political Efficacy (low vs. moderate) × Anger | -5.28 | 7.38 | -0.72 | .474 |
| External Political Efficacy (low vs. moderate) × Anger squared | 5.06 | 7.24 | 0.70 | .485 |
| External Political Efficacy (low vs. moderate) × Anger cubed | 1.58 | 7.21 | 0.22 | .826 |
| *Note*. Political efficacy is an ordinal variable (low and moderate; high external efficacy excluded from analysis because no variance in voting intention for several of the anger ranges at high external political efficacy such that nearly all participants reported intending to vote). Logistic regression coefficients are reported. Gender is coded as men = 0. Race is coded as Participants of Color = 0. Social class is coded as higher social class contexts = 0. Political party is coded as Democrat = 0. | | | | |

**S7 Table. Full model output for voting intention and internal political efficacy**

| **Model Parameter** | **B** | **SE** | ***z*** | ***p*** |
| --- | --- | --- | --- | --- |
| Internal Political Efficacy (linear) | 2.08 | 0.47 | 4.39 | < .001 |
| Internal Political Efficacy (quadratic) | -0.11 | 0.33 | -0.32 | .751 |
| Anger | -8.17 | 7.42 | -1.10 | .271 |
| Anger squared | -5.29 | 7.22 | -0.73 | .464 |
| Anger cubed | -6.72 | 7.02 | -0.96 | .339 |
| Anxiety intensity | 0.006 | 0.007 | 0.93 | .355 |
| Age | 0.04 | 0.01 | 2.78 | .005 |
| Gender (non-binary) | 9.77 | 882.74 | 0.01 | .991 |
| Gender (woman) | -0.44 | 0.39 | -1.13 | .259 |
| Race (White) | -0.58 | 0.42 | -1.38 | .169 |
| Social Class (lower) | -0.46 | 0.46 | -1.00 | .319 |
| Political Party (Republican) | 1.14 | 0.43 | 2.68 | .007 |
| Time | -0.02 | 0.02 | -1.23 | .218 |
| Internal Political Efficacy (linear) × Anger | -9.16 | 14.12 | -0.65 | .517 |
| Internal Political Efficacy (quadratic) × Anger | 0.98 | 10.15 | 0.10 | .923 |
| Internal Political Efficacy (linear) × Anger squared | 8.04 | 14.45 | 0.56 | .578 |
| Internal Political Efficacy (quadratic) × Anger squared | 3.61 | 10.31 | 0.35 | .726 |
| Internal Political Efficacy (linear) × Anger cubed | 13.72 | 13.92 | 0.99 | .324 |
| Internal Political Efficacy (quadratic) × Anger cubed | 0.95 | 9.96 | 0.10 | .924 |
| *Note*. Efficacy is an ordinal variable (low, moderate, and high). Logistic regression coefficients are reported. Gender is coded as men = 0. Race is coded as Participants of Color = 0. Social class is coded as higher social class contexts = 0. Political party is coded as Democrat = 0. | | | | |
