# Supplementary material for: From outrage to action: Efficacy moderates the relationship between anger and prosocial political outcomes
Source: PLoS One. 2026 Oct 14;21(10):e0358939. doi: 10.1371/journal.pone.0358939 (PMC13667767; doi:10.1371/journal.pone.0358939)
Supplement: Supplementary file 7 [file pone.0358939.s007.docx]

**S7 Table. Full model output for voting intention and internal political efficacy**

| **Model Parameter** | **B** | **SE** | ***z*** | ***p*** |
| --- | --- | --- | --- | --- |
| Internal Political Efficacy (linear) | 2.08 | 0.47 | 4.39 | < .001 |
| Internal Political Efficacy (quadratic) | -0.11 | 0.33 | -0.32 | .751 |
| Anger | -8.17 | 7.42 | -1.10 | .271 |
| Anger squared | -5.29 | 7.22 | -0.73 | .464 |
| Anger cubed | -6.72 | 7.02 | -0.96 | .339 |
| Anxiety intensity | 0.006 | 0.007 | 0.93 | .355 |
| Age | 0.04 | 0.01 | 2.78 | .005 |
| Gender (non-binary) | 9.77 | 882.74 | 0.01 | .991 |
| Gender (woman) | -0.44 | 0.39 | -1.13 | .259 |
| Race (White) | -0.58 | 0.42 | -1.38 | .169 |
| Social Class (lower) | -0.46 | 0.46 | -1.00 | .319 |
| Political Party (Republican) | 1.14 | 0.43 | 2.68 | .007 |
| Time | -0.02 | 0.02 | -1.23 | .218 |
| Internal Political Efficacy (linear) × Anger | -9.16 | 14.12 | -0.65 | .517 |
| Internal Political Efficacy (quadratic) × Anger | 0.98 | 10.15 | 0.10 | .923 |
| Internal Political Efficacy (linear) × Anger squared | 8.04 | 14.45 | 0.56 | .578 |
| Internal Political Efficacy (quadratic) × Anger squared | 3.61 | 10.31 | 0.35 | .726 |
| Internal Political Efficacy (linear) × Anger cubed | 13.72 | 13.92 | 0.99 | .324 |
| Internal Political Efficacy (quadratic) × Anger cubed | 0.95 | 9.96 | 0.10 | .924 |
| *Note*. Efficacy is an ordinal variable (low, moderate, and high). Logistic regression coefficients are reported. Gender is coded as men = 0. Race is coded as Participants of Color = 0. Social class is coded as higher social class contexts = 0. Political party is coded as Democrat = 0. | | | | |
